# Obstetric Violence in Spain (Part I): Women’s Perception and Interterritorial Differences

**DOI:** 10.3390/ijerph17217726

**Published:** 2020-10-22

**Authors:** Desirée Mena-Tudela, Susana Iglesias-Casás, Víctor Manuel González-Chordá, Águeda Cervera-Gasch, Laura Andreu-Pejó, María Jesús Valero-Chilleron

**Affiliations:** 1 Department of Nursing, Faculty of Health Sciences, Universitat Jaume I, Avda. Sos I Baynat s/n, 12071 Castellón, Spain; dmena@uji.es (D.M.-T.); vchorda@uji.es (V.M.G.-C.); pejo@uji.es (L.A.-P.); mjvalero.en@gmail.com (M.J.V.-C.); 2 Department of Obstetrics, Hospital do Salnés, Villgarcía de Aurousa, 36619 Pontevendra, Spain; matronasu@gmail.com

**Keywords:** obstetric violence, interterritorial differences, Spain, nursing, midwife, sexual and reproductive health

## Abstract

The decentralization of health systems can have direct repercussions on maternity care. Some inequalities can be noted in outcomes, like neonatal and child mortality in Spain. This study aimed to make the presence of obstetric violence in Spain visible as an interterritorial equity criterion. A descriptive, restrospective and cross-sectional study was conducted between January 2018 and June 2019. The sample comprised 17,541 questionnaires, which represented all Spanish Autonomous Communities. Of our sample, 38.3% perceived having suffered obstetric violence; 44.4% perceived that they had undergone unnecessary and/or painful procedures, of whom 83.4% were not requested to provide informed consent. The mean satisfaction with the attention women received obtained 6.94 points in the general sample and 4.85 points for those women who viewed themselves as victims of obstetric violence. Spain seems to have a serious problem with public health and respecting human rights in obstetric violence. Offering information to women and requesting their informed consent are barely practiced in the healthcare system, so it is necessary to profoundly reflect on obstetric practices with, and request informed consent from, women in Spain.

## 1. Introduction

No definition of Obstetric Violence (OV) as a result of a world consensus exists. Some countries have legislation about this concept and provide a definition [1]. In Spain, the OV Observatory defines such violence as “the act of ignoring the authority and autonomy that women have over their sexuality, their bodies, their babies and their pregnancy/birth experiences […], and of also ignoring the spontaneity, postures, rhythms and times that birth requires to progress normally; it is also the act of disregarding the emotional needs of both mother and baby at any time during pregnancy, while giving birth and during the immediate postpartum period” [2].

Regarding this concept, in 2014 the World Health Organization (WHO) defined which kinds of disrespectful and offensive treatments represented considerable lack of respect, which included the following: “physical abuse, profound humiliation and verbal abuse, coercive or unconsented medical procedures (including sterilization), lack of confidentiality, failure to get fully informed consent, refusal to give pain medication, gross violations of privacy, refusal of admission to health facilities, neglecting women during childbirth to suffer life-threatening, avoidable complications, and detention of women and their newborns in facilities after childbirth due to inability to pay” [3].

In 2015, the International Federation of Gynecology and Obstetrics (FIGO), along with the WHO, the International Confederation of Midwives (ICM), the International Pediatric Association (IPA) and the White Ribbon Alliance (WRA), proposed the Mother and Baby Friendly Birth Facility (MBFBF). This initiative is based on seven categories identified as: disrespect and abuse (Physical abuse; Non-consented care; Non-confidential care; Non-dignified care; Discrimination based on specific patient attributes; Abandonment of care and Detention in facilities). With this initiative, these organizations propose a series of criteria and indicators to facilitate classifying a healthcare institution as being apt for attending mothers and newborns [4].

In July 2019, the UN General Assembly issued an important report that indicated the situation of violence against women in reproductive healthcare services, which particularly emphasized attention at birth and OV [5]. This report stresses mobilizations taking place in many countries against such abusive practices. It proposes adopting an approach based on human rights to the various forms of mistreatment that women have suffered in the obstetric context by insisting on not only violating women’s rights to live a violence-free life, but also endangering their right to life, health, their physical integrity, their intimacy, their autonomy and their right against discrimination. Obstetric violence seems to be widespread throughout the world, with figures ranging from 18.3% [6] to 75.1% [7].

Nowadays, OV still exists without being accepted by most of the medical community, and even by society. The different types of OV detected in the literature refer to verbal violence, physical violence, sexual violence, social discrimination, neglect of care and inappropriate use of procedures and technologies [8]. The structural characteristic of such violence [9] often means that the professionals who exercise it are unaware that they do so, and even consider it to be normal [10,11]. Another factor to bear in mind is institutional violence. Such violence occurs when the healthcare administration does not spend enough human or material resources to ensure safe attention during deliveries and births [3]. From this institutional violence perspective, we can understand OV as an even broader concept in which political and legal factors can be found, where OV can be considered violence in which women cannot obtain sufficiently long maternity leave to attend to their babies’ physical and emotional needs, and to be able to offer their offspring, if they so wish, exclusive breastfeeding in the first six months of life in line with what the WHO and UNICEF recommend [12]. Within this framework, perhaps policies are also lacking, which need to be made to adapt the labor world to women’s requirements while pregnant or when raising their children, plus overcome the lack of efficient measures that favor real work-family conciliation.

We understand that any healthcare system intends to improve its population’s health [13]. Among a healthcare system’s main characteristics, we find each citizen’s right to protect his/her health, public financing and rendering services to ensure quality health care [14]. To this end, different existing healthcare system models will be used. In Spain, the law on the cohesion and quality of the National Health System (NHS) [15] allowed the political decentralization of health into its Spanish Autonomous Communities (SACs) in 2003. This decentralization of healthcare systems can have direct repercussions on maternity health care, a matter that has been the center of debate for several decades [16]. For instance, some studies report the benefits of such decentralization in settings with very few resources [17]. Some inequalities have also been found in outcomes, such as neonatal and child mortality, with differences in the outcomes of, and attention per SAC, in Spain [18]. Despite this decentralization taking place in Spain, Clinical Practice Guidelines are still issued by the Spanish Health Ministry for different medical conditions. One specific example of such dates back to 2010 when the Clinical Practice Guidelines for Normal Birth were published [19], which aimed to reduce variability in clinical practice by contributing to transforming the birth attention model in the healthcare system into more efficient, safer and more personalized attention for Spain [19]. Unifying criteria, even by considering the decentralization of the healthcare system, seems to offer positive results in the USA [20]. No studies have been found in Spain that evidence variability of clinical practice in relation to maternity and OV after the Spanish healthcare system had been decentralized. This is why the present study aims to make the presence of OV in Spain visible as an interterritorial equity criterion.

## 2. Materials and Methods

### 2.1. Design, Population and Sample

A descriptive, retrospective and cross-sectional study was carried out between January 2018 and June 2019. The study population was formed by women attended to in a Spanish public or private hospital to give birth naturally or by cesarean section, or for miscarriage. Women who were treated in the period from 2009 to 2018 and who responded to the questionnaire were included in the study. The exclusion criteria were: giving birth at home or at a hospital outside Spain, and not completing 80% of the questionnaire or more. Those questionnaires from the SAC of Ceuta and Melilla were also excluded for being poorly representative, as were the questionnaires in which the province item was not answered. During this period in Spain, the Clinical Practice Guidelines for Normal Birth were implemented [19]. The study was designed in accordance with the principles of the Declaration of Helsinki (charity, no maleficence, autonomy and justice) and with Spanish Organic Law 03/2018 on Protection of Personal Data and Guarantee of Digital Rights. No personal data, IP or email address, which could compromise participants’ identity, was collected, and answering the questionnaire implied giving consent. Participants were informed of these aspects before voluntarily answering the questionnaire.

### 2.2. Data Collection

Data collection was carried out between February and April 2018 using an online ad hoc questionnaire, which was sent to healthcare professionals, child rearing groups, breastfeeding support groups, administrators of blogs and the association «Birth is Ours» [21], by sending the questionnaire link via social networks like WhatsApp or Facebook [22,23]. These associations, groups and health professionals were responsible for distributing the questionnaire to the women.

The main socio-demographic variable was the province and SAC that women belonged to when they were pregnant, gave birth and during their puerperium period. The other variables were: received healthcare attention (public health care, private health care, or mixed) and satisfaction with the attention received (on a visual analogical scale from 1 (not at all satisfied) to 10 (extremely satisfied). Variables were added about the perceived support from healthcare institutions related to pregnancy, birth, puerperium and breastfeeding rights; information received about the process they experienced; criticisms about their behavior; being treated with childish diminutives; not being able to resolve fears or doubts; unnecessary interventions; respect for birth plan; breastfeeding support; perceived having suffered OV. These variables were measured as: Yes, No, Do not know/no answer. Finally, some questions were added about gestational loss and the support received wherever appropriate, and about women’s overall opinion of the healthcare attention they received: (a) empowered and satisfied; (b) insecure, vulnerable, guilty, incapable; (c) indifferent; (d) do not know/no answer.

### 2.3. Statistical Analysis

Data were processed with the Statistical Package program for Social Sciences (SPSS) v. 25, IBM, Armonk, NK, USA. A descriptive analysis of all the variables was performed with frequency, percentage or mean, standard deviation, maximum and minimum according to the nature of the variable. The relation between satisfaction and the received attention in interterritorial terms was studied with a one-factor ANOVA.

A hierarchal cluster analysis was done following the Ward method by considering the Euclidean distance to group the SAC according to how the surveyed women perceived OV. Finally, a bivariate analysis was carried out using the Chi squared test using contingency tables or a one-factor ANOVA, as necessary. To examine interterritorial differences, the groups provided by the cluster analysis were employed. Statistical significance was set at *p* < 0.05.

## 3. Results

In all, 17,742 questionnaires were obtained, of which 201 were eliminated (1.13%): 88 (0.49%) because they were completed by women who had given birth abroad or because they were not properly filled in; 17 (0.09%) because they belonged to the SAC Ceuta and Melilla; 96 (0.54%) because they did not include an answer to the province variable. The final sample included 17,541 questionnaires.

The descriptive data indicated that 65.3% (*n* = 11,450) of the women had been attended to by public health care, 24.3% (*n* = 4261) by a mixed combination of public–private health care and the rest of the population was treated in a private hospital (10.4%, *n* = 1830). Table 1 shows the sample distribution into SAC.

**Figure 1:**
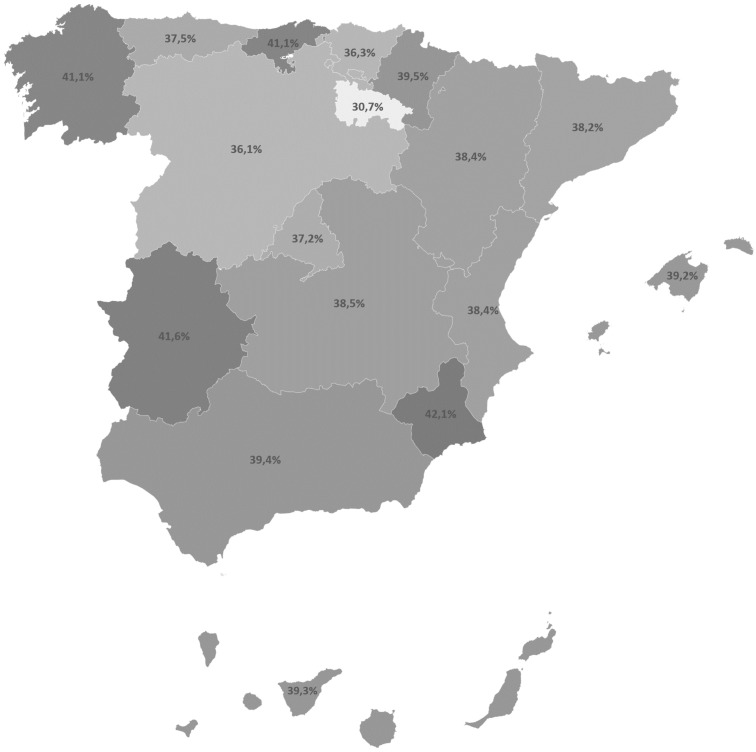
Frequency of Obstetric Violence (OV) per SAC.

In regard to how women perceived having suffered OV during pregnancy, while giving birth or in the postpartum period, 38.3% (*n* = 6051) of our sample indicated having suffered OV. Figure 1 shows how the OV percentages are distributed into SAC. The darker the colors in this figure, the more positive women’s answers about OV were.

**Figure 2:**
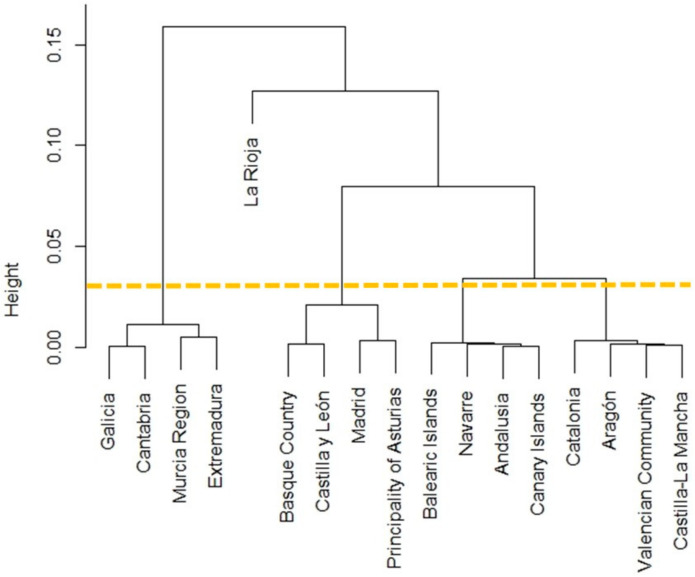
Dendrogram showing the conclusive groups obtained by the cluster analysis.

Figure 2 is a dendrogram of the cluster analysis per SAC, with which five groups were obtained. Group 1 was formed by SAC Madrid, Basque Country, Principality of Asturias and Castilla y León (mean percentage = 36.77%; Interquartile Range (IQR) = 1.04); Group 2 was made up of SAC Catalonia, Valencian Community, Aragón and Castilla-La Mancha (mean percentage = 38.37%; IQR = 0.15); Group 3 comprised SAC Andalusia, Balearic Islands, Canary Islands and Navarre (mean percentage = 39.35%; IQR = 0.08); Group 4 consisted of SAC Murcia Region, Galicia, Extremadura and Cantabria (mean percentage = 41.46%; IQR = 0.57); the last group included only one SAC: La Rioja (mean percentage = 30.68%). Statistically significant differences among cluster groups were confirmed with (*X*^2^ = 16.97; *df* = 4; *p* = 0.002). OV occurred mainly in the private health care context (*p* = 0.023) (Table 2).

### 3.1. Obstetric Violence Related to Health Professionals’ Behavior

Of all the surveyed women, 67.9% (*n* = 10,664) believed that healthcare institutions (Ministry of Health, Health Services, Management, Regional Ministries) did not sufficiently support or promote their rights for pregnancy, birth, puerperium and breastfeeding; 45.9% (*n* = 8047) of the sample indicated that they were not informed about the procedures they had been submitted to, nor were they asked to provide express consent; 34.5% (*n* = 6045) stated that they were criticized for their behavior by means of ironic or discrediting remarks; 31.4% (*n* = 5502) had been treated with nicknames or childish diminutives; 48.0% (*n* = 8423) indicated that they found it impossible to resolve their doubts, or to voice their fears or concerns; 44.4% (*n* = 7786) perceived that they had undergone unnecessary and/or painful procedures, and of these, 52.3% (*n* = 4026) were neither provided with reasons nor asked to give consent, and 31.1% (*n* = 2406) were provided with reasons, but were not asked to give consent. Thus, a total of 83.4% (*n* = 6432) were not requested to provide informed consent. Table 3 offers the bivariate analysis of the variables related to health professionals’ behavior according to the cluster groups, and highlights that those variables that revealed no statistically significant differences for groups were the ones involved in requesting informed consent (*p* > 0.05). 

### 3.2. Obstetric Violence Related to Birth Plan

With the variables related to birth plan, 41.6% (*n* = 6833) of the women reported not having received information about an existing birth plan. Therefore, 53.3% (*n* = 8771) of the cases did not deliver their birth plan and only 20.2% (*n* = 3323) of the women thought that their birth plan was respected. Table 4 shows the bivariate analysis as cluster groups.

### 3.3. Obstetric Violence Related to Miscarriage and Perinatal Loss

Of all cases, 6.3% (*n* = 1108) of pregnancies ended in miscarriage or perinatal loss, and no statistically significant differences were found in the cluster groups (*X*^2^ = 2.96; *df* = 4; *p* = 0.565). Of those women who suffered miscarriage or perinatal loss, 36.5% (*n* = 404) perceived unnecessary or unjustified care. Once again, no statistically significant differences appeared in the cluster groups (*X*^2^ = 4.13; *df* = 4; *p* = 0.389). Moreover, 70.8% (*n* = 785) of the cases did not feel they had received physiological support during the process (*X*^2^ = 9.62; *df* = 4; *p* = 0.047).

### 3.4. Obstetric Violence Related to Postpartum and Breastfeeding

Of all the participants, 35.0% (*n* = 5751) answered that they did not feel they received any support during the postpartum in the questions about feed and baby care. Of those who chose breastfeeding, 37.6% (*n* = 6123) did not feel they were supported or helped to resolve doubts or overcome difficulties (Table 5).

### 3.5. Satisfaction with the Attention Received

As for feeling satisfied with the attention received, we obtained a mean score of 6.94 points (SD ± 2.517, Max. = 10; Min. = 1). The attention received for 54.5% (*n* = 9569) of the cases made women feel insecure, vulnerable, guilty, incapable or indifferent (Table 6).

The mean score for feeling satisfied with the attention received for those women who reported having suffered OV was 4.85 points (*n* = 6051; SD = 2.367; 95%CI = 4.79–4.91), while the women who did not report OV obtained a mean score of 8.31 points (*n* = 9732; SD = 1.622; 95%CI = 8.28–8.34) (*p* < 0.001).

## 4. Discussion

The present study analyzed the presence of OV in Spain as an interterritorial equity criterion. To do so, a cluster analysis was used to classify the 17 SACs in Spain into five different groups according to the level of OV referred to by the women in the sample. Mapping was also done to show the distribution of the women who perceived having suffered OV in each SAC.

We must stress that the theoretical revision of the OV concept as a recognized phenomenon indicates that such violence may occur during pregnancy, birth and the puerperium, and in situations such as miscarriage, postmiscarriage and the reproductive cycle [8]. The first relevant finding was that more than 38% of the surveyed women assured having suffered OV while being attended to during pregnancy, birth or the puerperium. Although no works were found in the literature that report similar figures among Spanish women, some studies were encountered and indicate the proportion of OV in other countries, like Ethiopia with 75.1% [7], Brazil with 18.3% [6] or India with 28.8% [24]. These different results might be due to differences in each study’s sample sizes [7], and also to discrepancies in the various instruments employed to assess such violence. In Europe, OV is a theme that is increasingly at the center of debate, and is particularly promoted by organizations and different social movements to defend human rights [25]. Perhaps this is one of the reasons why very few studies can be found in Europe. Nonetheless, one study conducted with Italian women concluded that the proportion of women who felt they had been victims of OV was 21.2% [26]. This notably lower percentage is comparable to the figures found in our study as Italy has more socio-economic and geographic similarities to Spain than the other countries found in the scientific literature. Other European countries have reported similar figures to the Italian ones [27]. Nonetheless, the high percentage obtained in Spain is not surprising because in recent years the UN has urged Spain to directly fight against OV [28], but it is still necessary to explore the reasons for such violence in Spain.

About private health care in Spain, different reports suggest that its protocols are more obsolete, with less humanization shown while giving birth and the most attention is paid to the physical aspect of delivery, while emotional and psychological aspects are neglected [19,29]. Moreover, the rates at which interventions are made in private health care while giving birth (e.g., induced birth, instrumentalized birth or cesarean section) can sometimes triple those applied in Spanish public health care [29,30]. Almost 10 years ago, The Spanish Ministry of Health urged private health care to provide more transparent statistics about indicators of attending delivery and maternity, and not only cesarean section rates [31]. However, this request continues to be ignored. So it is not surprising that OV is more related to private health care, just as the present study discovered and which can be seen reflected in Cluster 4, made up of the autonomous communities of Murcia Region, Galicia, Extremadura and Cantabria. Future studies should relate the various obstetric interventions made to the OV level perceived by the women attended to by both public and private health cares.

According to the distribution of both the mapping and cluster groups found by the analysis, the La Rioja SAC obtained the lowest percentage for OV: it formed a single group in the cluster analysis, and was found to be a pioneer insofar as it has implemented its own normal birth attention strategy, which is periodically assessed [32]. These results sharply contrast with other key NHS indicators as La Rioja was one of the SACs with the highest perinatal mortality rate in the Spanish NHS during our study period [30]. This finding is a surprising one because neonatal mortality is more closely related to the quality of attention and is less sensitive to differences in social class [18]. Moreover, and what is absolutely congruent with the found cluster groups, among the key indicators the cesarean section rates apparently denote a certain relation to these groups [30], which should be confirmed in future studies. Given that the World Health Organization includes in its definition avoidable complications resulting from disrespect and abuse during childbirth, future lines of research could also detect complications of obstetric interventions associated to OV.

Three relevant aspects came over from how women perceived health professionals’ treatment: support perceived by institutions for women’s rights; direct treatment with women; offering women information and asking them for informed consent.

Regarding institutions’ support for women’s rights, different works appear in the literature which support the notion that OV violates human rights and is a serious public health problem [8]. According to the jurisprudence about sexual and reproductive rights, and international law, OV violates the right to health, intimacy, no discrimination, no violence and no torture, among others [33]. To be able to defend women from such violence, Latin America is the world region that has coined the legal term OV with specific legislation in different countries [1], although legislating and making OV criminal have not had the expected positive effect on recognizing women’s reproductive rights [34]. In Europe, legislation on OV is still a pending matter, so it is necessary to empower more vulnerable women as to their human and their sexual-reproductive rights [35], and to offer them tools to defend themselves against invisible or socially accepted actions [36].

As for treatment with women, in the present study roughly one third of the women stated having been criticized for their behavior and treated with childish diminutives. Almost half of the women were unable to resolve their doubts about, or voice their fears of, the process they were going through. Other studies have found similar results for this matter [37,38]; it is stressed that women end up accepting going through procedures without asking questions, and without voicing their desires and their doubts [8]. Women not feeling confident about the healthcare system and the professionals attending them, of their fear of or the vulnerability of the process they are going through, seem to back these results which, according to the present results, also directly affects their satisfaction with the attention they received [39,40].

Offering women information and asking them for informed consent seem practices that have rarely been set up in a generalized manner in health care. Here it is worth pointing out that the structural dimension of OV [9] seems more tangible in this variable because even in the SACs that practice less OV, this information and requesting express consent do not occur in over 40% of cases. Similar results have been found in the reviewed literature [7,8,41,42]. Reflecting on obstetric practices and asking women for informed consent, who feel completely dominated by the healthcare staff’s technical–scientific authority and the patriarchal authority of structural violence, is necessary. Denying women the right to make informed decisions about the health care they receive while giving birth is a violation of their human rights [5].

Finally, regarding the support women perceived during the postpartum and for breastfeeding, the results indicate that the cluster group which presented less OV referred to more breastfeeding support. This support is reflected in the breastfeeding rates shown for the various SACs, where La Rioja had a similar breastfeeding starting rate to the other SACs, but presented a much higher breastfeeding percentage at six months than other communities [43]. The international literature has also related the presence of OV with little received support to commence breastfeeding [44]. Indeed mistreatment, abuse and OV are found to influence women’s decision making and their capacity to breastfeed their babies. These findings must make those organizations in charge of public health policy making reflect on the advantage of breastfeeding because it is a fundamental aspect of materno-infant health, and must be considered to form part of women’s sexual-reproductive lives, rather than being separated from the integral attention offered by the healthcare system.

This study has attempted to deal with and well-represent interterritorial differences in OV in Spain. Even so, it is not without some limitations, which must be contemplated when interpreting its results. First, it is necessary to consider that this is a retrospective study based on how women perceived OV. Hence memory and information biases could be present, without forgetting that childbirth is a vital part of women’s sexual-reproductive lives, and many of its aspects are engraved on their memory [45]. A non-probabilistic sampling was carried out and this may affect the representativeness of the sample. In addition, there was a possibility of selection bias, as the questionnaire was distributed to groups that may be more sensitive to the subject matter of the study. It should be noted that variables such as age, socioeconomic and cultural variables, number of children or date of birth were not collected to allow a descriptive socio-demographic analysis to be carried out to allow comparison with other populations. It is stressed that the decentralization concept and the type of health care offered in Spain shape an almost unique healthcare model, which means that some of its results cannot be extrapolated to other health systems. It is also necessary to highlight that after decentralization, all the SACs have possibly offered different legal and management healthcare policies to regulate this competence, which could have influenced the obtained results and should be contemplated in future studies.

## 5. Conclusions

What the present study seems to reflect is that Spain has a serious public health problem and one of respecting human rights in relation to OV. The fact that the health system has been decentralized in SACs and their respective healthcare models may have influenced their outcomes, it is necessary to consider the major differences in how OV is perceived by the various analyzed groups. It is also necessary to contemplate that private health care can act as a context that is more predisposed to OV in Spain. Action protocols and training for health personnel must continue to be updated and put into practice, as must transparency policies that contribute real data about how women are treated in these centers.

It is fundamental to stress lack of confidence, fear or typical vulnerability of some processes in the perinatal stage, which mean that women do not voice their doubts about, or fears of, interventions. This means that women may unconsciously allow OV to perpetuate. So reflecting on obstetric practices and asking women for informed consent, who feel completely dominated by the healthcare staff’s technical–scientific authority and the patriarchal authority of structural violence, is necessary.

Finally, we must be aware of, and invest in, improving some indicators related to maternal and infant health, such as breastfeeding. To do so, it is important to consider all maternity healthcare aspects regardless of them emerging during pregnancy, birth and the puerperium, and in certain situations like miscarriage, postmiscarriage and the reproductive cycle, as part of women’s sexual-reproductive lives. We should not disassociate the integral attention that the health system offers or the legislation, which should ideally exist to protect women from OV.

## Figures and Tables

**Table 1:** Sample distribution into Spanish Autonomous Community (SAC) (*n* = 17,541).

Spanish Autonomous Community	*n*	%
Madrid	4101	23.4
Catalonia	1882	10.7
Valencian Community	1410	8.0
Andalusia	1780	10.1
Murcia Region	236	1.3
Basque Country	1039	5.9
Balearic Islands	314	1.8
Canary Islands	507	2.9
Galicia	1933	11.0
Principality of Asturias	649	3.7
Aragón	1054	6.0
Castilla-La Mancha	774	4.4
Extremadura	342	1.9
Navarre	247	1.4
Cantabria	119	0.7
Castilla y León	1060	6.0
La Rioja	94	0.5
Total	17,541	100

**Table 2:** Descriptive data about the variable perceiving Obstetric Violence according to the cluster group and the type of attention received (*n* = 15783).

	Perceived Obstetric Violence							
Cluster Group	Total		Public		Private		Mixed	
	Yes% (*n*)	No% (*n*)	Yes% (*n*)	No% (*n*)	Yes% (*n*)	No% (*n*)	Yes% (*n*)	No% (*n*)
1	36.9 (2277)	63.1 (3891)	43.0 (1677)	57.0 (2226)	53.0 (337)	47.0 (299)	16.1 (263)	83.9 (1366)
2	38.3 (1773)	61.7 (2851)	43.6 (1316)	56.4 (1705)	54.0 (272)	46.0 (232)	16.8 (185)	83.2 (914)
3	39.4 (1010)	60.6 (1556)	44.7 (749)	55.3 (925)	61.5 (171)	38.5 (107)	14.7 (90)	85.3 (524)
4	41.2 (964)	58.8 (1373)	46.1 (701)	53.9 (821)	61.6 (180)	38.4 (112)	15.9 (83)	84.1 (440)
5	30.7 (27)	69.3 (61)	40.8 (20)	59.2 (29)	41.7 (5)	58.3 (7)	7.4 (2)	92.6 (25)
***X*** **^2^**	16.97		5.07		11.39		2.90	
***df*** **^1^**	4		4		4		4	
***p*** **^2^**	0.002		0.280		0.023		0.574	

^1^*df*: Degrees of Freedom; ^2.^ Chi-squared test.

**Table 3:** The bivariate results of the variables related to health professionals’ behavior.

		Cluster Group								
		Total	1	2	3	4	5	*X* ^2^	*df* ^1^	*p* ^2^
Rights Supported and Promoted by Healthcare Institutions								34.41	8	<0.001
Yes	*n*	3484	1459	980	554	464	27			
	%	22.2	23.7	21.7	21.6	19.4	30.3			
No	*n*	10,664	4073	3053	1795	1689	54			
	%	67.9	66.2	67.6	69.9	70.8	60.7			
NK/NA ^3^	*n*	1567	623	484	218	234	8			
	%	10.0	10.1	10.7	8.5	9.8	9.0			
Patient Being Asked for Express Consent and Provided with Information								14.86	8	0.062
Yes	*n*	9093	3653	2612	1469	1305	54			
	%	51.8	53.3	51.0	51.6	49.6	57.4			
No	*n*	8047	3044	2386	1318	1260	39			
	%	45.9	44.4	46.6	46.3	47.9	41.5			
NK/NA ^3^	*n*	401	152	122	61	65	1			
	%	2.3	2.2	2.4	2.1	2.5	1.1			
Patient Criticized by Ironic or Discrediting Remarks								22.57	8	0.004
Yes	*n*	6045	2307	1737	992	984	25			
	%	34.5	33.7	33.9	34.8	37.4	26.6			
No	*n*	11,203	4443	3285	1814	1594	67			
	%	63.9	64.9	64.2	63.7	60.6	71.3			
NK/NA ^3^	*n*	293	99	98	42	52	2			
	%	1.7	1.4	1.9	1.5	2.0	2.1			
Treated with Nicknames or Childish Diminutives								15.97	8	0.043
Yes	*n*	5502	2068	1613	939	859	23			
	%	31.4	30.2	31.5	33.0	32.7	24.5			
No	*n*	9867	3936	2895	1541	1438	57			
	%	56.3	57.5	56.5	54.1	54.7	60.6			
NK/NA ^3^	*n*	2172	845	612	368	333	14			
	%	12.4	12.3	12.0	12.9	12.7	14.9			
Difficult or Impossible to Voice Doubts, Fears or Concerns								15.72	8	0.047
Yes	*n*	8423	3200	2465	1384	1332	42			
	%	48.0	46.7	48.1	48.6	50.6	44.7			
No	*n*	8618	3449	2517	1374	1227	51			
	%	49.1	50.4	49.2	48.2	46.7	54.3			
NK/NA ^3^	*n*	500	200	138	90	71	1			
	%	2.9	2.9	2.7	3.2	2.7	1.1			
Patient Perceived Unnecessary and/or Painful Procedures During Birth								23.81	8	0.002
Yes	*n*	7786	2930	2318	1281	1223	34			
	%	44.4	42.8	45.3	45.0	46.5	36.2			
No	*n*	8675	3494	2511	1365	1250	55			
	%	49.5	51.0	49.0	47.9	47.5	58.5			
NK/NA ^3^	*n*	1080	425	291	202	157	5			
	%	6.2	6.2	5.7	7.1	6.0	5.3			
Patient Asked for Consent for Unnecessary and/or Painful Procedures								7.36	8	0.498
Yes	*n*	487	183	127	98	75	4			
	%	6.3	6.3	5.6	7.6	6.3	11.4			
No	*n*	6959	2636	2061	1153	1079	30			
	%	90.4	90.4	91.3	89.0	90.4	85.7			
NK/NA ^3^	*n*	251	97	69	44	40	1			
	%	3.3	3.3	3.1	3.4	3.4	2.9			

^1^*df*: Degrees of Freedom; ^2^ Chi-squared test; ^3^ NK/NA: Not know/no answer.

**Table 4:** The bivariate analysis results of the variables related to birth plan.

		Cluster Group								
		Total	1	2	3	4	5	*X* ^2^	*df* ^1^	*p* ^2^
Information Received about Birth Plan								22.82	12	0.029
Yes	*n*	6940	2725	2063	1097	1008	47			
	%	42.2	42.3	43.1	41.1	41.2	52.2			
No	*n*	6833	2713	1919	1102	1067	32			
	%	41.6	42.1	40.0	41.2	43.6	35.6			
NK/NA ^3^	*n*	541	215	162	85	76	3			
	%	3.3	3.3	3.4	3.2	3.1	3.3			
I was informed, but my doubts were not resolved	*n*	2127	785	648	388	298	8			
	%	12.9	12.2	13.5	14.5	12.2	8.9			
Respecting Birth Plan								35.73	16	0.003
Yes	*n*	3323	1299	971	518	516	19			
	%	20.2	20.2	20.3	19.4	21.1	21.1			
I delivered no birth plan	*n*	8771	3568	2485	1381	1286	51			
	%	53.3	55.4	51.9	51.7	52.5	56.7			
No, but I was given reasons	*n*	1736	642	545	293	248	8			
	%	10.6	10.0	11.4	11.0	10.1	8.9			
No, and I was not given any reasons	*n*	2115	752	641	384	329	9			
	%	12.9	11.7	13.4	14.4	13.4	10.0			
NK/NA ^3^	*n*	497	178	150	96	70	3			
	%	3.0	2.8	3.1	3.6	2.9	3.3			

^1^*df*: Degrees of Freedom; ^2^ Chi-squared Test; ^3^ NK/NA: Not know/no answer.

**Table 5:** The bivariate analysis results of the variables related to the postpartum and breastfeeding.

		Cluster Group								
		Total	1	2	3	4	5	*X* ^2^	*Df* ^1^	*p* ^2^
Support During Postpartum								37.76	8	<0.001
Yes	*n*	10,027	4042	2957	1565	1400	63			
	%	61.0	62.8	61.7	58.6	57.2	70.0			
No	*n*	5751	2154	1629	991	953	24			
	%	35.0	33.5	34.0	37.1	38.9	26.7			
NK/NA ^2^	*n*	664	243	206	116	96	3			
	%	4.0	3.8	4.3	4.3	3.9	3.3			
Breastfeeding Support								46.91	8	<0.001
Yes	*n*	9077	3696	2660	1414	1250	57			
	%	55.7	57.8	56.1	53.5	51.5	63.3			
No	*n*	6123	2276	1778	1066	972	31			
	%	37.6	35.6	37.5	40.3	40.1	34.4			
NK/NA ^3^	*n*	1096	426	302	163	203	2			
	%	6.7	6.7	6.4	6.2	8.4	2.2			

^1^*df*: Degrees of Freedom; ^2^ Chi-squared Test; ^3^ NK/NA: Not know/no answer.

**Table 6:** Satisfaction with the attention received per cluster group (*n* = 17,541).

	Satisfied with the Attention Received				The Attention Received Made You Feel …							
Cluster	*n*	Mean	SD ^1^	95%CI ^2^	Empowered, Satisfied		Insecure, Vulnerable, Guilty, Incapable		Indifferent		NK/NA ^5^	
					*n*	%	*n*	%	*n*	%	*n*	%
1	6849	7.05	2.477	6.99–7.11	2790	40.7	2436	35.6	1150	16.8	472	6.9
2	5120	6.93	2.535	6.86–7.00	2011	39.3	1856	36.3	920	18.0	33	6.5
3	2848	6.87	2.539	6.77–6.96	1048	36.8	1085	38.1	174	6.1	541	19.0
4	2630	6.70	2.555	6.60–6.79	892	33.9	1050	39.9	479	18.2	208	7.9
5	94	7.48	2.184	7.03–7.93	38	40.4	30	31.9	22	23.4	4	4.3
***F***	11.31				***X^2^***	54.82						
***df ^3^***	4				***df***	12						
***p***	<0.001 ^4^				***p***	<0.001 ^6^						

^1^ SD: Standard deviation. ^2^ 95%CI: 95% confident interval; ^3^ *df*: Degrees of Freedom; ^4^ one-factor ANOVA; ^5^ NK/NA: Not know/no answer; ^6^ Chi-squared test.
